# Supplementary material for: Social isolation among mothers caring for Infants in Japan: findings from the Nationwide Survey of healthy parents and children 21
Source: Matern Child Health J. 2022 Apr 18;26(7):1549–58. doi: 10.1007/s10995-022-03427-0 (PMC9174307; doi:10.1007/s10995-022-03427-0)
Supplement: Supplementary file 1 — Supplementary Material 1 [file 10995_2022_3427_MOESM1_ESM.docx]

Appendix 1. Differences Between Included and Excluded Participants

|  | Included | |  | Excluded | | *P** | (df) |
| --- | --- | --- | --- | --- | --- | --- | --- |
|  | *n* | % |  | *n* | % |  |  |
| **Independent Variable: Child-Rearing Isolation** | |  |  |  |  |  |  |
| **Child-Rearing Isolation** |  |  |  |  |  |  |  |
| No | 69,177 | 99.8 |  | 3,416 | 99.4 | < .001 | (1) |
| Yes | 160 | 0.2 |  | 19 | 0.6 |  |  |
|  |  |  |  |  |  |  |  |
| **Explanatory Variables** |  |  |  |  |  |  |  |
| **Maternal Age at Childbirth** |  |  |  |  |  |  |  |
| ≤ 24 years | 7,625 | 11.0 |  | 1,272 | 21.4 | < .001 | (4) |
| 25–29 years | 20,726 | 29.9 |  | 1,678 | 28.2 |  |  |
| 30–34 years | 24,576 | 35.4 |  | 1,747 | 29.4 |  |  |
| 35–39 years | 14,137 | 20.4 |  | 1,000 | 16.8 |  |  |
| ≥ 40 years | 2,273 | 3.3 |  | 243 | 4.1 |  |  |
|  |  |  |  |  |  |  |  |
| **Child Age** |  |  |  |  |  |  |  |
| 3–4 months | 19,474 | 28.1 |  | 1,255 | 20.0 | < .001 | (2) |
| 18 months | 25,522 | 36.8 |  | 2,400 | 38.2 |  |  |
| 3 years | 24,341 | 35.1 |  | 2,630 | 41.8 |  |  |
|  |  |  |  |  |  |  |  |
| **Mothers' Employment Status** |  |  |  |  |  |  |  |
| Employed | 36,014 | 51.9 |  | 2,398 | 64.2 | < .001 | (1) |
| Unemployed | 33,323 | 48.1 |  | 1,335 | 35.8 |  |  |
|  |  |  |  |  |  |  |  |
| **Socioeconomic Status** |  |  |  |  |  |  |  |
| Average or above average | 47,562 | 68.6 |  | 2,072 | 56.0 | < .001 | (1) |
| Difficult | 21,775 | 31.4 |  | 1,628 | 44.0 |  |  |
|  |  |  |  |  |  |  |  |
| **Husband's Child-Rearing Participation** |  |  |  |  |  |  |  |
| Yes | 62,645 | 90.3 |  | 1,493 | 88.8 | .030 | (1) |
| Almost none | 6,692 | 9.7 |  | 189 | 11.2 |  |  |
|  |  |  |  |  |  |  |  |
| **Living Municipality** |  |  |  |  |  |  |  |
| City | 54,337 | 78.4 |  | 5,090 | 81.0 | < .001 | (3) |
| City designated by government ordinance | 3,762 | 5.4 |  | 242 | 3.9 |  |  |
| Town/village | 10,009 | 14.4 |  | 882 | 14.0 |  |  |
| Special ward (Tokyo ward) | 1,229 | 1.8 |  | 71 | 1.1 |  |  |
|  |  |  |  |  |  |  |  |
| **Time to Relax with Child** |  |  |  |  |  |  |  |
| Yes | 48,942 | 70.5 |  | 2,453 | 66.6 | < .001 | (1) |
| No | 20,395 | 29.2 |  | 1,228 | 33.4 |  |  |
|  |  |  |  |  |  |  |  |
| **Child-Rearing Confidence** |  |  |  |  |  |  |  |
| Confident | 20,168 | 29.1 |  | 1,124 | 31.7 | .001 | (1) |
| Not confident | 49,169 | 70.9 |  | 2,425 | 68.3 |  |  |
|  |  |  |  |  |  |  |  |
| **Concerns about Abusing Own Child** |  |  |  |  |  |  |  |
| No | 52,431 | 75.6 |  | 2,597 | 74.5 | .126 | (1) |
| Yes | 16,906 | 24.4 |  | 890 | 25.5 |  |  |
| *Note*. Differences in all variables between the included and excluded ones were estimated using the χ^2^ tests  **p* < .05 | | | | | | | |

**p* < .05

Appendix 2. Results of Logistic Regression Analysis with "Isolation of Child-Rearing" as the Objective Variable (MICE)

|  |  | Multivariate analysis | | | | |
| --- | --- | --- | --- | --- | --- | --- |
|  |  | OR |  | 95% CI |  | *P** |
| **Maternal Age at Childbirth** |  |  |  |  |  |  |
| ≤ 24 years |  | 1.2 |  | [0.7, 2.0] |  | .554 |
| 25–29 years |  | 1.3 |  | [0.9, 1.9] |  | .210 |
| 30–34 years |  |  |  |  |  |  |
| 35–39 years |  | 1.6 |  | [1.0, 2.4] |  | .029 |
| ≥ 40 years |  | 2.2 |  | [1.1, 4.4] |  | .019 |
|  |  |  |  |  |  |  |
| **Child Age** |  |  |  |  |  |  |
| 3–4 months |  |  |  |  |  |  |
| 18 months |  | 1.0 |  | [0.6, 1.6] |  | .999 |
| 3 years |  | 1.6 |  | [1.0, 2.5] |  | .033 |
|  |  |  |  |  |  |  |
| **Mothers' Employment Status** | |  |  |  |  |  |
| Employed |  |  |  |  |  |  |
| Unemployed |  | 1.7 |  | [1.2, 2.3] |  | .001 |
|  |  |  |  |  |  |  |
| **Socio-Economic Status** |  |  |  |  |  |  |
| Average or above average |  |  |  |  |  |  |
| Difficult |  | 1.9 |  | [1.4, 2.5] |  | < .001 |
|  |  |  |  |  |  |  |
| **Husband's Participation in Child-Rearing** | | | | |  |  |
| Yes |  |  |  |  |  |  |
| Almost none |  | 5.6 |  | [4.1, 7.8] |  | < .001 |
|  |  |  |  |  |  |  |
| **Living Municipality** |  |  |  |  |  |  |
| City |  |  |  |  |  |  |
| City designated by  government ordinance | | 1.2 |  | [0.7, 2.2] |  | .552 |
| Town/village |  | 1.0 |  | [0.6, 1.5] |  | .874 |
| Special ward (Tokyo ward) |  | 3.9 |  | [2.0, 7.5] |  | < .001 |
|  |  |  |  |  |  |  |
| **Time to Relax with Child** |  |  |  |  |  |  |
| Yes |  |  |  |  |  |  |
| No |  | 4.8 |  | [3.3, 7.0] |  | < .001 |
|  |  |  |  |  |  |  |
| **Child-Rearing Confidence** |  |  |  |  |  |  |
| Confident |  |  |  |  |  |  |
| Not confident |  | 1.4 |  | [0.9, 2.3] |  | .169 |
|  |  |  |  |  |  |  |
| **Concerns about Abusing Own Child** | | |  |  |  |  |
| No |  |  |  |  |  |  |
| Yes |  | 2.0 |  | [1.4, 2.7] |  | < .001 |

**p* < .05

Appendix 3. Frequencies of Complete and Imputed Variables

|  | Complete | Imputed | Total |
| --- | --- | --- | --- |
| **Maternal Age at Childbirth** | 75,277 | 345 | 75,622 |
|  |  |  |  |
| **Child Age** | 75,622 | 0 | 75,622 |
|  |  |  |  |
| **Mothers' Employment Status** | 73,070 | 2,552 | 75,622 |
|  |  |  |  |
| **Socio-Economic Status** | 73,037 | 2,585 | 75,622 |
|  |  |  |  |
| **Husband's Participation in Child-Rearing** | 71,019 | 4,603 | 75,622 |
|  |  |  |  |
| **Living Municipality** | 75,622 | 0 | 75,622 |
|  |  |  |  |
| **Time to Relax with Child** | 73,018 | 2,604 | 75,622 |
|  |  |  |  |
| **Child-Rearing Confidence** | 72,886 | 2,736 | 75,622 |
|  |  |  |  |
| **Concerns about Abusing Own Child** | 72,824 | 2,798 | 75,622 |
